# Letter from the Editor in Chief

**DOI:** 10.19102/icrm.2018.090707

**Published:** 2018-07-15

**Authors:** Moussa Mansour


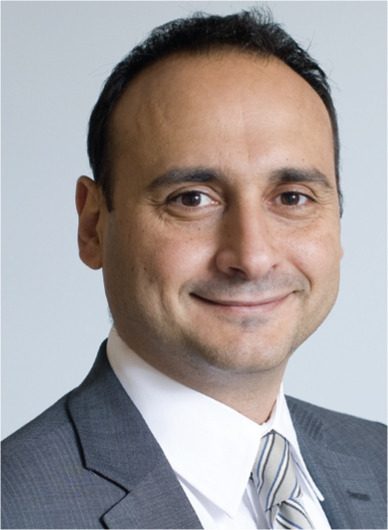


Dear Readers,

This issue of *The Journal of Innovations in Cardiac Rhythm Management* contains an important article by Kohno et al.^1^ titled “Trends in Subcutaneous Cardiac Monitoring Technology.” In it, the authors focus on the status of implantable cardiac monitors and discuss their indications, limitations, and future applications.

This article is important because it highlights the growing field of ambulatory cardiac monitoring, which has exploded in the past few years. Novel monitoring devices, both wearable and implantable, have been introduced and have been used extensively. New indications for monitoring have also been described, and monitoring devices are now being prescribed for a wide variety of conditions. The evolution of this field has been fueled primarily by the growing epidemic of atrial fibrillation and the expansion of its treatments. Long-term cardiac monitoring after cryptogenic stroke and following catheter ablation are two recent practices that were not widely done a few years ago but which are now being performed commonly. Let’s also not forget about the growing interest in detecting undiagnosed atrial fibrillation in patients at risk of developing this disease, a practice that is being investigated.^2^

At the current time, the clear majority of cardiac monitoring is being performed by health care providers, using medical devices—such as Holter monitors, event recorders, or implantable cardiac monitors like the Confirm Rx™ (Abbott Laboratories, Chicago, IL, USA) or Reveal LINQ™ (Medtronic, Minneapolis, MN, USA) devices—prescribed as part of a comprehensive diagnostic and treatment plan for patients. However there has also been an emergence of the use of consumer devices for the detection of heart rhythm disorders. Some of these consumer devices, which include KardiaMobile and KardiaBand (both AliveCor, San Francisco, CA, USA) and Beat2Phone (VitalSignum Oy, Helsinki, Finland), provide high-quality recordings comparable to those of prescribed medical devices. The technology overall is rapidly advancing and I believe that, soon, devices such as smartwatches and low-profile wearable tools with direct communication to smartphones will be widely used by the general population to detect all arrhythmias. However, one caveat to mention is that, while the hardware aspect of this technological revolution has been rapidly evolving, the software part appears to be lagging. Specifically, advanced tools that automatically detect abnormal rhythms do not seem to be highly accurate at this stage. As such, because of the potential for a sharp increase in the use of consumer devices for cardiac monitoring, it is critically important to use machine learning and artificial intelligence to develop algorithms that accurately detect cardiac rhythm disorders and filter out the vast majority of the normal recordings.

I hope that you enjoy reading this issue of *The Journal of Innovations in Cardiac Rhythm Management*. Best wishes for a relaxing summer.

Sincerely,


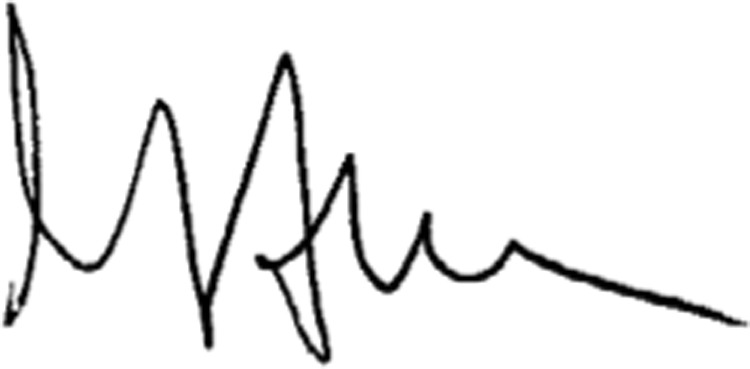


Moussa Mansour, MD, FHRS, FACC

Editor in Chief

The Journal of Innovations in Cardiac Rhythm Management

MMansour@InnovationsInCRM.com

Director, Atrial Fibrillation Program

Jeremy Ruskin and Dan Starks Endowed Chair in Cardiology

Massachusetts General Hospital

Boston, MA 02114
